# Identification of a novel HLA-A^*^02:01-restricted cytotoxic T lymphocyte epitope derived from the EML4-ALK fusion gene

**DOI:** 10.3892/or.2014.3198

**Published:** 2014-05-19

**Authors:** MAYUKO YOSHIMURA, YOSHITAKA TADA, KAZUYA OFUZI, MASAKAZU YAMAMOTO, TETSUYA NAKATSURA

**Affiliations:** 1 Division of Cancer Immunotherapy, Exploratory Oncology Research and Clinical Trial Center, National Cancer Center, Kashiwa, Chiba 277-8577, Japan; 2 Department of Gastroenterological Surgery, Tokyo Women’s Medical University, Shinzyukuku, Tokyo 162-8666, Japan; 3 Research Institute for Biomedical Sciences, Tokyo University of Science, Chiba 278-0022, Japan

**Keywords:** EML4-ALK, peptide vaccine, CTL clone, lung cancer

## Abstract

Cancer immunotherapy is a promising new approach to cancer treatment. It has been demonstrated that a high number of tumor-specific cytotoxic T cells (CTLs) is associated with increased disease-specific survival in lung cancer patients. Identification of superior CTL epitopes from tumor antigens is essential for the development of immunotherapy for malignant tumors. The EML4-ALK fusion gene was recently identified in a subset of non-small cell lung cancers (NSCLCs). In this study we searched for HLA-A^*^02:01- and HLA-A^*^24:02-restricted epitopes derived from EML4-ALK by screening predicted EML4-ALK-derived candidate peptides for the induction of tumor-reactive CTLs. Nine EML4-ALK-derived peptides were selected by a computer algorithm based on a permissive HLA-A^*^02:01 or HLA-A^*^24:02 binding motif. One of the nine peptides induced peptide-specific CTLs from human peripheral blood mononuclear cells. We were able to generate a peptide-specific CTL clone. This CTL clone specifically recognized peptide-pulsed T2 cells and H2228 cells expressing HLA-A^*^02:01 and EML4-ALK that had been treated with IFN-γ 48 h prior to examination. CTL activity was inhibited by an anti-HLA-class I monoclonal antibody (W6/32), consistent with a class I-restricted mechanism of cytotoxicity. These results suggest that this peptide (RLSALESRV) is a novel HLA-A^*^02:01-restricted CTL epitope and that it may be a new target for antigen-specific immunotherapy against EML4-ALK-positive cancers.

## Introduction

Lung cancer is one of the main causes of cancer-related mortality. Approximately 85% of lung cancers are diagnosed as non-small cell lung cancer (NSCLC), and the overall survival (OS) rate for advanced NSCLC is poor. The 5-year survival rate is 5% for stage IIIb NSCLC and <1% for stage IV NSCLC (1). Treatment for NCSLC is determined by the patient’s clinical and tumor characteristics, performance status (PS), the histological subtype and tumor genotype/phenotype.

Recently, there have been many studies concerning agents that target molecular changes, such as mutations in the epidermal growth factor receptor (EGFR) and the fusion oncogene EML4-ALK, in which the echinoderm microtubule-associated protein-like 4 (EML4) is fused with the intracellular domain of anaplastic kinase (ALK) (2–4). Although significant advances have been made in the treatment of NSCLC using molecular targeted therapies such as erlotinib and crizotinib, the median OS for patients with advanced NSCLC remains low (5,6), and acquired resistance to target agents is a major clinical problem. Therefore, the development of novel therapies is needed (7).

Immunotherapy manipulates the immune system to control and eradicate cancer. Many recent studies provide evidence suggesting that immunotherapeutic manipulations are viable in many tumor types, including lung cancer. Numerous trials of peptide vaccines, autologous cellular therapy, T cell-directed antibody therapy and monoclonal antibody therapy for lung cancer have been carried out around the world (8–10) and some of them have shown favorable results (11–13).

The EML4-ALK fusion gene was identified in NSCLC patients by a team led by Professor H. Mano. This fusion gene was formed as the result of a small inversion within the short arm of chromosome 2 that joins differing portions of the EML4 gene with a portion of the ALK gene (14,15). As a result of this fusion, constant dimerization of the kinase domain of ALK is induced and its catalytic activity increases consequently.

The EML4-ALK fusion gene is mainly identified in young, never/former light smokers with NSCLC (16). It is estimated that approximately 5% of all NSCLC cases have this fusion gene. A few reports have also identified EML4-ALK in other cancers, namely breast cancer and colorectal cancer (17,18). For the most part, the EML4-ALK fusion gene and other mutations, such as those in EGFR and KRAS, are mutually exclusive (19).

The chromosomal inversion does not always occur in the same location, and multiple EML4-ALK variants have been identified (19). At least 11 variants have been reported. The most common variants are E13;A20 (variant 1) and E6a/b;A20 (variant 3a/b), which have been detected in 33% and 29% of NSCLC patients, respectively (14).

PF-02341066 (crizotinib) is an ALK inhibitor currently under clinical development. Kwak *et al* conducted an open-label, multi-center, two-part phase I trial and found a remarkable 57% overall response rate and a 72% 6-month progression-free survival rate (20).

In spite of the marked antitumor activity of crizotinib, ALK-positive cancers invariably gain resistance to crizotinib. In the case of ALK-positive cancers, as well as EGFR-mutant lung cancer, resistance develops on average within the first 2 years of therapy (21). The main resistance mutations are L1196M, a gatekeeper mutation and C1156M. In addition to ALK mutations, other known mechanisms for acquired resistance include ALK amplification (21,22) and EGFR activation (23,24). To overcome resistance, new ALK inhibitors are currently in early phase studies (25). Novel combinatorial strategies to overcome crizotinib resistance and further improve the clinical outcome are needed.

We focused on this new fusion array as a novel target of immunotherapy. There are several methods to detect EML4-ALK NSCLC, including polymerase chain reaction (PCR), immunohistochemistry (IHC) and fluorescence in situ hybridization (FISH) (19). These methods detect high-level EML4-ALK fusion gene expression. Passoni *et al* identified two HLA-A^*^02:01-restricted ALK-derived peptides that induce peptide-specific CTL lines (26).

We focused on the EML4 array as a novel epitope of immunotherapy. We identified a candidate 9- or 10-amino acid array of novel epitopes using the Bioinformatics and Molecular Analysis Section (BIMAS) software and analyzed its potential as a new immunotherapy epitope, with respect to its ability to induce anticancer activity. We then induced and generated a peptide-specific CTL clone from peripheral blood lymphocytes of HLA-A^*^02:01-positive healthy donors. We report here that an EML4-ALK-derived peptide-specific human CTL clone recognized peptide-pulsed T2 cells and HLA-A^*^02:01-positive and EML4-ALK-positive tumor cells pretreated with IFN-γ. Furthermore, we showed that immunotherapy with this novel epitope peptide has potential for treatment of EML4-ALK-positive NSCLC.

## Materials and methods

### Peptides

Human EML4-ALK-derived peptides carrying binding motifs for HLA-A^*^02:01-/HLA-A^*^24:02-encoded molecules were identified by HLA-peptide binding predictions using the BIMAS program (http://bimas.dcrt.nih.gov/molbio/hla_bind/index.html). We purchased a total of seven EML4-ALK-derived peptides carrying HLA-A^*^02:01 binding motifs and two peptides carrying HLA-A^*^24:02 binding motifs from Geneworld (Tokyo, Japan).

### Cell lines

The H2228 human lung adenocarcinoma cell line and EML4-ALK fusion protein variant 3 (E6; A20) were kindly provided by Professor S. Yano (Kanazawa University).

T2 is a lymphoblastoid cell line that lacks TAP function and has HLA-A^*^02:01 molecules that can easily be loaded with exogenous peptides. T2A24 is the same cell line but with HLA-A^*^24:02 instead. T2 and T2A24 cells were cultured in RPMI medium supplemented with 10% heat-inactivated FBS.

### HLA-A^*^02:01/HLA-A^*^24:02 binding assay

In order to determine the binding ability of the predicted peptides to HLA-A^*^02:01/HLA-A^*^24:02 molecules, an *in vitro* cellular binding assay was performed as reported previously (27).

Briefly, after incubation of the T2/T2A24 cells in culture medium at 26°C for 18 h, cells were washed with PBS and suspended in 1 ml Opti-MEM (Invitrogen, Carlsbad, CA, USA) with or without 100 μg peptide and then incubated at 26°C for 3 h and at 37°C for 3 h. After washing with PBS, HLA-A^*^02:01/HLA-A^*^24:02 expression was measured by flow cytometry using a FITC-conjugated and HLA-A^*^02:01-/HLA-A^*^24:02-specific monoclonal antibody (mAb) and the mean fluorescence intensity was recorded.

### Generation of dendritic cells

CD14^+^ cells were isolated from human peripheral blood mononuclear cells (PBMCs) using human CD14 microbeads (Miltenyi Biotec, Bergisch Gladbach, Germany). Immature dendritic cells (DCs) were generated from CD14^+^ cells using interleukin (IL)-4 (10 ng/ml; PeproTech Inc., Rocky Hill, NJ, USA) and granulocyte-macrophage colony-stimulating factor (GM-CSF; 10 ng/ml; PeproTech) in RPMI-1640 medium supplemented with 10% FBS. Maturation of DCs was induced by prostaglandin E2 (PGE2; 1 μg/ml; Sigma, St. Louis, MO, USA) and tumor necrosis factor (TNF-)-α (10 ng/ml; PeproTech).

### Induction of EML4-ALK-derived peptide-specific CTLs from PBMCs

CD8^+^ cells were isolated from PBMCs using human CD8 microbeads (Miltenyi Biotec, Bergisch Gladbach, Germany). CD8^+^ cells (2×10^6^) were stimulated by peptide-pulsed irradiated autologous mature DCs (1×10^5^). Autologous DCs were prepared from a limited supply; artificial antigen presenting cells (aAPCs) (K562/A2 or A24/CD80/CD83) were alternatively used for further examination. After 1 week, these cells were stimulated twice per week by peptide-pulsed irradiated artificial APC-A2 or artificial APC-A24 cells (1×10^5^). Supplementation with 10 IU/ml IL-2 (Proleukin; Novartis Pharmaceuticals, Basel, Switzerland) and 10 ng/ml IL-15 (PeproTech) was performed every 3 to 4 days between stimulations (28).

### IFN-γ ELISPOT assay

Specific secretion of IFN-γ from human CTLs in response to stimulator cells was assayed using the IFN-γ ELISPOT kit (BD Biosciences), according to the manufacturer’s instructions. Stimulator cells were pulsed with peptide for 2 h at room temperature and then washed. Responder cells were incubated with stimulator cells for 20 h. The resulting spots were counted using an ELIPHOTO counter (Minerva Tech, Tokyo, Japan). HIV-gag (77–85) (SLYNTYATL) was used as an irrelevant peptide in the CTL assay.

### Generation of CTL clones

Cultured cells were incubated with peptide-pulsed T2/T2A24 cells at a ratio of 2:1 for 3.5 h at 37°C. CD107a-specific antibodies (BioLegend, San Diego, CA, USA) were included in the mixture during the incubation period. CD8^+^CD107a^+^ cells were sorted using a FACSAria II cell sorter (BD Biosciences). Sorted CTLs were stimulated and the CTL clones were established as described previously (29).

### Flow cytometry

H2228 cells with or without pretreatment with 100 U/ml IFN-γ (PeproTech) for 48 h were harvested and stained with anti-HLA-A2 Ab-FITC (MBL, Japan) and analyzed using a FACSCanto II flow cytometer (BD Biosciences). Flow cytometry data were analyzed using FlowJo software.

### Cytotoxicity assay

The cytotoxic capacity was analyzed using the Terascan VPC system (Minerva Tech, Tokyo). The CTL clone was used as the effector cell type. Target cells treated with 100 U/ml IFN-γ (PeproTech) 42 h previously were labeled through incubation in calcein-AM solution for 30 min at 37°C. The labeled cells (1×10^4^) were then co-cultured with the effector cells for 4–6 h. Fluorescence intensity was measured before and after the culture period, and specific cytotoxic activity was calculated as described previously (29).

HLA-A^*^02:01 blocking of T-cell activity was tested by pre-incubating the target cells with anti-HLA-A, -B, -C mAb (W6/32) or an isotype control mAb (mIgG2a,κ; BioLegend San Diego, CA, USA).

## Results

### Identification of HLA-A^*^02:01-/HLA-A^*^24:02-restricted EML4-ALK-derived peptides

**Figure 1:**
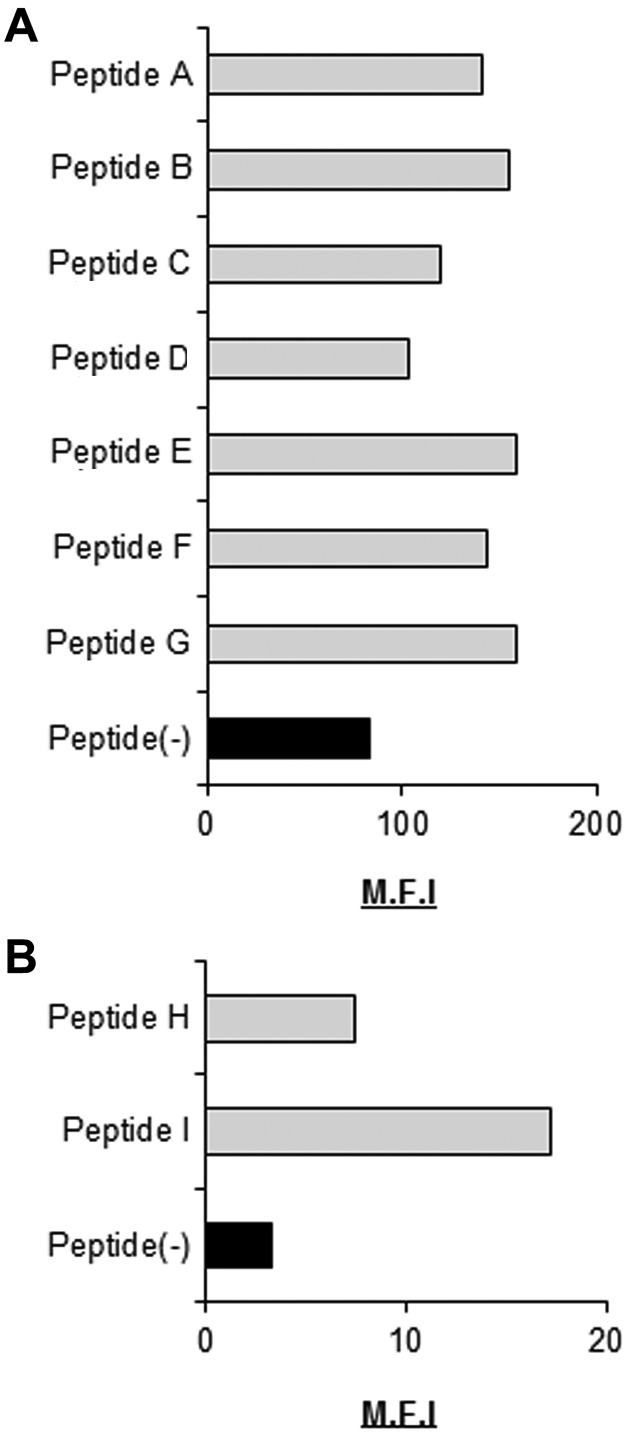
EML4-ALK-derived peptides bound to HLA-A2 or HLA-A24 molecules. *In vitro* cellular peptide binding assays for HLA-A^*^02:01 (A) or HLA-A^*^24:02 (B) were performed using a FACS system.

As candidate EML4-ALK- derived and HLA-A^*^02:01-/HLA-A^*^24:02-restricted CTL epitopes, we selected nine peptides with highly predicted scores for HLA-A^*^02:01/HLA-A^*^24:02 binding calculated using BIMAS software (Tables I and II) and evaluated their ability to bind to HLA-A^*^02:01/HLA-A^*^24:02 molecules. All nine peptides were able to bind HLA-A^*^02:01/HLA-A^*^24:02 molecules (Fig. 1).

### Generation of an EML4-ALK-derived peptide-specific CTL clone from human PBMCs

**Figure 2:**
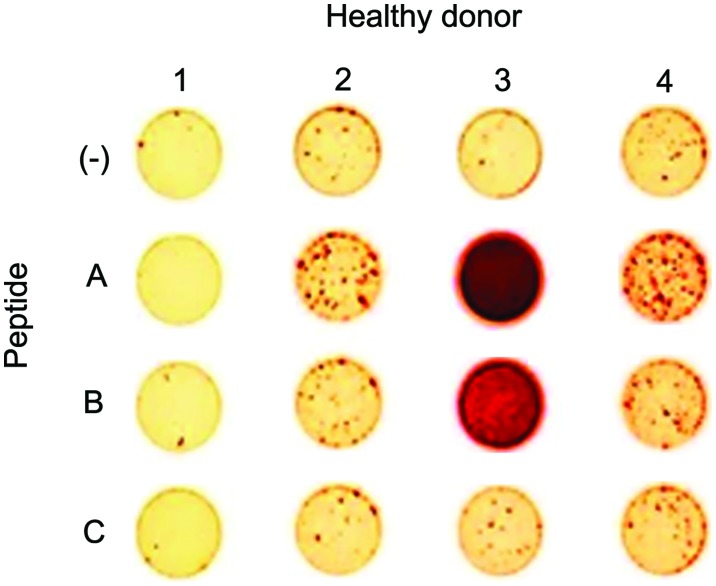
IFN-γ release by *in vitro*-induced anti-EML4-ALK CTLs. CD8^+^ T cells from four healthy donors were stimulated with EML4-ALK-derived peptide-pulsed autologous DCs and aAPCs. CTLs induced by EML4-ALK-derived peptides (1×10^5^) were stimulated with T2 cells pulsed with or without 1 μM EML4-ALK-derived peptides. IFN-γ-producing CTLs were detected by IFN-γ ELISPOT assay. DCs, dendritic cells; aAPCs, artificial antigen presenting cells; CTLs, cytotoxic T cells.

We next assessed the capacity of EML4-ALK-derived peptides to generate peptide-specific CTLs *in vitro* from human PBMCs of HLA-A^*^02:01/HLA-A^*^24:02 healthy donors. CTLs were induced by three stimulations with DCs or artificial APCs loaded with the EML4-ALK-derived peptides. CTLs were tested for specificity for each peptide using the IFN-γ ELISPOT assay. Peptides A, B and C could induce peptide-specific CTLs that were able to specifically recognize T2 cells pulsed with each peptide, but not T2 cells without peptides (Fig. 2). Peptides B and C were able to induce CTLs from only one donor (healthy donor 3 for peptide B and healthy donor 4 for peptide C), but peptide A was able to induce CTLs in three of four donors (healthy donors 2, 3 and 4). Based on this result, we used peptide A for further examinations.

**Figure 3:**
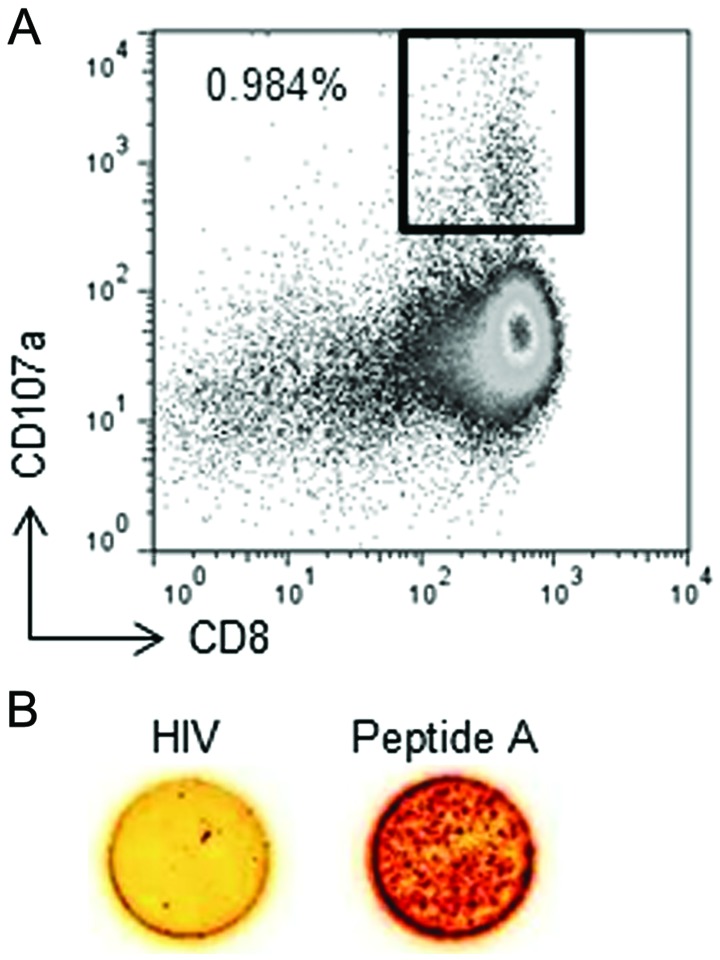
Peptide A-specific CTL clone established from anti-EML4-ALK CTL. (A) Peptide A-specific CTL clones established using CD107a single cell sorting. Peptide A-specific CTLs (1×10^5^) were incubated with peptide-pulsed T2 cells (5×10^4^) with CD107a-specific antibodies for 3.5 h at 37°C. CD8^+^CD107a^+^ cells were sorted using a FACSAria II cell sorter. Square, CD8^+^CD107a^+^ cells that are peptide A-specific CTL clones. (B) Recognition of peptide-pulsed T2 cells by peptide A-specific CTL clones. A peptide A-specific CTL clone (1×10^4^ cells) was incubated with stimulator cells that had been pulsed with 1 μM peptide A or HIV-gag peptide. IFN-γ-producing CTLs were detected by IFN-γ ELISPOT assay. CTLs, cytotoxic T cells.

Next, we obtained one CTL clone from peptide A-specific CTLs that was able to specifically recognize T2 cells pulsed with peptide A, but not T2 cells pulsed with an irrelevant HIV-gag peptide, using single cell sorting with a CD107a antibody. The population of CD8^+^CD107a^+^ cells represented 0.984% of all stimulated cells (Fig. 3A). These cells were sorted as single cells in each well of a 96-well plate. Twenty-one days after cell sorting, peptide specificity was assessed using the IFN-γ ELISPOT assay (Fig. 3B). The established clone reacted to the T2 cells pulsed with peptide A, but not to T2 cells pulsed with the irrelevant HIV-gag peptide. These results indicate that a peptide A-specific CTL clone was successfully established from PBMCs from a healthy donor.

### The EML4-ALK-specific CTL clone recognizes HLA-A^*^02:01^+^ lung carcinoma cells with the EML4-ALK variant 3a/b incubated with IFN-γ

**Figure 4:**
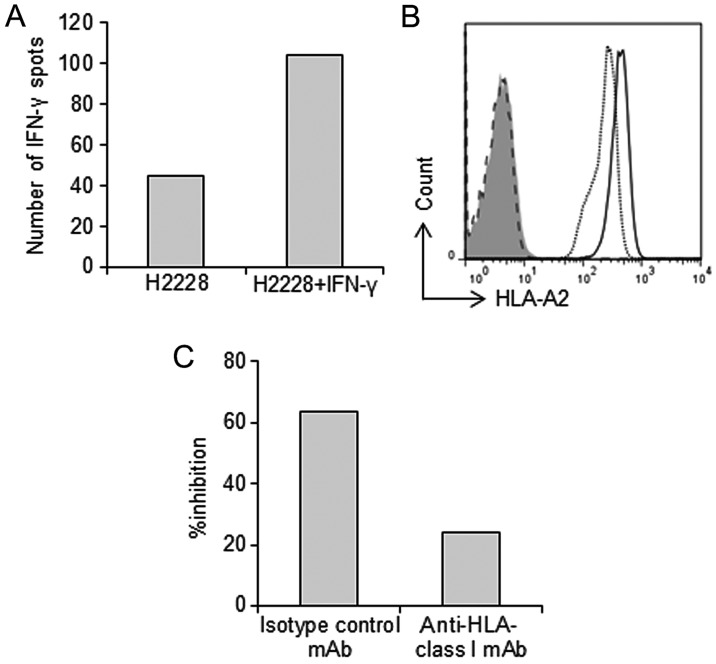
Recognition of lung carcinoma cells expressing HLA-A^*^02:01 and the EML4-ALK fusion gene by the peptide A-specific CTL clone. The peptide A-specific CTL clone recognized H2228 cells pretreated with IFN-γ 48 h prior to the assay. (A) The peptide A-specific CTL clone (1×10^4^ cells) was incubated with H2228 cells with or without IFN-γ. IFN-γ production was detected by IFN-γ ELISPOT assay. (B) IFN-γ increased expression of HLA-A2 presented on H2228 cells. Incubation of H2228 cells with 100 U/ml IFN-γ for 48 h increased HLA-A2 presentation on the cells. Dotted line, HLA-A2 on H2228 cells without IFN-γ. Black line, HLA-A2 on H2228 cells incubated with IFN-γ (higher than on H2228 cells without IFN-γ). Dashed line and shaded region: no staining of H2228 cells with/without IFN-γ. (C) Inhibition of IFN-γ production by an anti-HLA-class I mAb. Blocking experiments were performed using an HLA-A, -B, -C-specific mAb (W6/32) or an isotype control mAb (mIgG2a,κ). The peptide A-specific CTL clone was incubated with H2228 cells (HLA-A^*^02:01^+^/EML4-ALK^+^) pretreated with IFN-γ 48 h prior to examination. IFN-γ-producing CTL clones were detected by IFN-γ ELISPOT assay. The bar graph shows the percentage of inhibition. CTL, cytotoxic T cell.

We next evaluated the ability of the EML4-ALK-specific CTL clone to recognize the cancer cell line H2228, which expresses HLA-A^*^02:01 and EML4-ALK, using the IFN-γ ELISPOT assay. Even though the EML4-ALK-specific CTL clone failed to recognize H2228 cells, it did recognize those pretreated with 100 U/ml IFN-γ 48 h prior to examination (Fig. 4A). We examined the effect of IFN-γ on H2228 cells. Incubating target cells with IFN-γ for 48 h increased the expression of MHC class I molecules on the cell surface (Fig. 4B). This result indicates that the peptide A-specific CTL clone was able to recognize H2228 cells because of increased expression of MHC-class I on the H2228 cell surface.

Specific IFN-γ production by the peptide A-specific CTL clone was detectable in H2228 cells treated with IFN-γ. The specificity was abolished by an anti-HLA-class I mAb, but not by an isotype control, suggesting that the observed production was HLA-A2 restricted (Fig. 4C).

**Figure 5:**
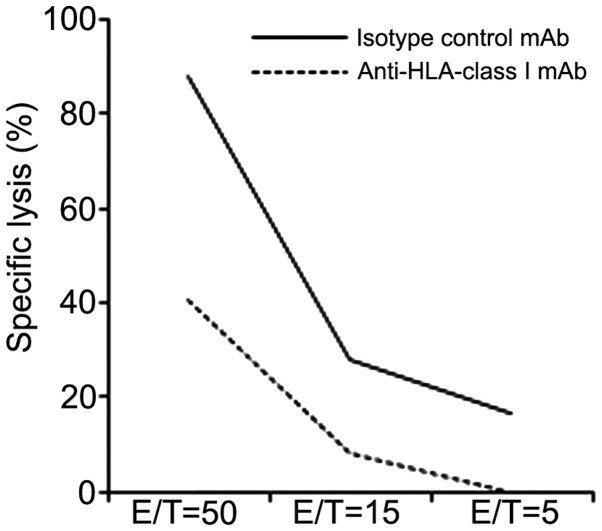
Cytotoxic activity of the peptide A-specific CTL clone against H2228 cells. The peptide A-specific CTL clone was incubated with H2228 cells pretreated with IFN-γ 48 h prior to the assay at various E/T ratios, and specific lysis was assessed. Blocking experiments were performed using the HLA-A, -B, -C-specific mAb (W6/32) or the isotype control mAb (mIgG2a,κ). CTL, cytotoxic T cell.

A cytotoxicity assay was also performed. The peptide A-specific CTL clone was able to specifically lyse H2228 cells pretreated with IFN-γ 48 h prior to examination. This specific lysis was blocked by the anti-HLA-class I mAb, but not by the isotype control. These results indicate that the peptide A-specific CTL clone showed cytotoxicity and the ability to produce IFN-γ against HLA-A^*^02:01^+^ EML4-ALK^+^ NSCLC cell lines (Fig. 5).

## Discussion

In the present study, we identified a new tumor-associated CTL epitope (peptide A) derived from EML4-ALK, which binds to HLA-A^*^02:01 molecules, and we were able to establish a peptide-specific CTL clone from human PBMCs that specifically recognized cognate peptide-pulsed T2 cells and HLA-A^*^02:01 tumor cells expressing EML4-ALK that had been pretreated with IFN-γ.

EML4-ALK-positive lung cancers are highly sensitive to ALK inhibition. However, as with trastuzumab or gefitinib (30,31), patients typically gain resistance within 1 to 2 years of starting therapy (23). We aimed to overcome these difficulties with immunotherapy.

We identified a glypican-3 (GPC3)-derived peptide and showed that GPC3-specific CTL frequency after vaccination correlated with OS. OS was significantly longer in patients with high GPC3-specific CTL frequencies than in those with low frequencies (32). This indicates that the ability to induce a peptide-specific CTL clone is important for effective immunotherapy. We also revealed that GPC3 is an ideal target for anticancer immunotherapy since it is specifically overexpressed in hepatocellular carcinoma (HCC) (33–35).

In the present study, we chose a peptide array from EML4-ALK, from which we were able to induce a peptide-specific CTL clone. EML4-ALK is a strong oncogene overexpressed in cancer cells of NSCLC, breast cancer, kidney cancer and colon cancer (17). We performed RT-PCR and assayed the EML4 DNA levels of certain lung cancer cell lines. H2228 cells express EML4 moderately but at higher levels than other lung cancer cell lines. EML4 expression has been reported as highly expressed in CD8^+^ T cells. RT-PCR showed that EML4 DNA levels were high in PBMCs and CD8^+^ T cells. Because of a lack of suitable antibodies, we could not perform western blotting. However, our success at inducing a peptide A-specific CTL clone from CD8^+^ T cells indicated that the CTL clone had no cytotoxicity against CD8^+^ T cells.

This CTL clone could not recognize cancer cell lines without the ability to increase the amount of HLA class I presented on cell surfaces. Further examination is needed to achieve higher tumor reactivity. Combination chemotherapy or radiation therapy plus immunotherapy was recently reported to have a synergistic effect (36). Moreover, some mechanisms of synergy between radiation therapy, chemotherapy and immunotherapy have been revealed (37). In one of the mechanisms, these therapies upregulated tumor antigens and MHC moieties. These results suggest that combination therapy could be used to make tumor cell lines more susceptible to this peptide A-specific CTL clone-mediated cytolysis (38–41).

In addition, this treatment may be able to overcome resistance to ALK inhibition. Some resistance mechanisms for targeting drugs have been examined. The most commonly identified causes of resistance are point mutations such as L1196M (42–44), G1269A (22) and S1206Y (21). These point mutations occur in the tyrosine kinase domain, which plays an important role in oncogenesis. Our peptide array was selected from EML4, which has no correlation with these point mutations. It is possible that this treatment is effective for tumor cells resistant to ALK inhibitors.

In this study, we identified a new epitope peptide derived from the EML4-ALK fusion gene. We successfully induced an HLA-A^*^02:01-restricted peptide-specific CTL clone that demonstrated cytotoxicity for EML4-ALK-positive tumor cells. This is a new epitope-based vaccine therapy design for EML4-ALK-positive cancer cells. In order to obtain a stronger effect, further analysis is needed.

## Figures and Tables

**Table I:** HLA-A2 peptide binding predictions of the BIMAS program.

Peptide name	Peptide sequence	Binding score^a^
A	RLSALESRV	69.552
B	AISEDHVASV	90.183
C	TVLKAALADV	51.79
D	KLIPKVTKT	59.989
E	YLLPTGEIV	237.82
F	MLIWSKTTV	118.238
G	VMLIWSKTTV	315.95

a Binding scores were estimated using BIMAS software (http://www-bimas.cit.nih.gov/mobio/hla_bind/).

**Table II:** HLA-A24 peptide binding predictions of the BIMAS program.

Peptide name	Peptide sequence	Binding score^a^
H	NYDDIRTEL	369.6
I	VYFIASVVVL	200

a Binding scores were estimated using BIMAS software (http://www-bimas.cit.nih.gov/mobio/hla_bind/).
